# Spinal Muscular Atrophy autophagy profile is tissue-dependent: differential regulation between muscle and motoneurons

**DOI:** 10.1186/s40478-021-01223-5

**Published:** 2021-07-03

**Authors:** Alba Sansa, Ivan Hidalgo, Maria P. Miralles, Sandra de la Fuente, M. Jose Perez-Garcia, Francina Munell, Rosa M. Soler, Ana Garcera

**Affiliations:** 1 Neuronal Signaling Unit, Experimental Medicine Department, Universitat de Lleida-IRBLleida, Rovira Roure, 80, 25198 Lleida, Spain; 2 Paediatric Neuromuscular Disorders Unit, Pediatric Neurology Group, Vall d’Hebron University Hospital and Vall d’Hebron Institute of Research (VHIR), Barcelona, Spain

**Keywords:** Spinal muscular atrophy, Survival motor neuron, Autophagy, Neuromuscular disease, Human iPSCs, Neurodegeneration, Motoneuron

## Abstract

Spinal muscular atrophy (SMA) is a neuromuscular genetic disease caused by reduced survival motor neuron (SMN) protein. SMN is ubiquitous and deficient levels cause spinal cord motoneurons (MNs) degeneration and muscle atrophy. Nevertheless, the mechanism by which SMN reduction in muscle contributes to SMA disease is not fully understood. Therefore, studies evaluating atrophy mechanisms in SMA muscles will contribute to strengthening current knowledge of the pathology. Here we propose to evaluate autophagy in SMA muscle, a pathway altered in myotube atrophy. We analized autophagy proteins and mTOR in muscle biopsies, fibroblasts, and lymphoblast cell lines from SMA patients and in gastrocnemius muscles from a severe SMA mouse model. Human MNs differentiated from SMA and unaffected control iPSCs were also included in the analysis of the autophagy. Muscle biopsies, fibroblasts, and lymphoblast cell lines from SMA patients showed reduction of the autophagy marker LC3-II. In SMA mouse gastrocnemius, we observed lower levels of LC3-II, Beclin 1, and p62/SQSTM1 proteins at pre-symptomatic stage. mTOR phosphorylation at Ser2448 was decreased in SMA muscle cells. However, in mouse and human cultured SMA MNs mTOR phosphorylation and LC3-II levels were increased. These results suggest a differential regulation in SMA of the autophagy process in muscle cells and MNs. Opposite changes in autophagy proteins and mTOR phosphorylation between muscle cells and neurons were observed. These differences may reflect a specific response to SMN reduction, which could imply diverse tissue-dependent reactions to therapies that should be taken into account when treating SMA patients.

## Introduction

Spinal muscular atrophy (SMA) is a genetic neuromuscular disorder characterized by progressive muscle weakness and atrophy [1]. In infants, SMA is the most common cause of death due to a genetic origin and affects 1 in 6,000 to 10,000 live births [2]. SMA is initiated by deficient levels of the Survival Motor Neuron (SMN) protein, which is codified by the *SMN* genes [3]. In humans there are two versions of *SMN*, *SMN1* gene responsible for the production of full-length SMN protein (ubiquitously expressed), and several copies of *SMN2* gene that suffers alternative splicing and produces predominantly a short version of SMN lacking exon 7 [4, 5]. When *SMN1* is absent by mutation, deletion, or conversion, *SMN2* is not able to compensate for the loss of *SMN1*.

Spinal cord motoneurons (MNs) loss during SMA is a hallmark of the disease, together with severe muscle atrophy [1]. Muscle-specific studies in SMA suggest deleterious changes in muscle tissue previous to MN degeneration [6] and recent results showed that selective depletion of SMN in muscle tissue reveals MN-independent disease [7]. However, the role of muscle cells in the SMA pathology is not entirely understood. Intracellular processes related to protein turnover regulation may contribute to muscle defects observed in SMA. Autophagy is a highly regulated pathway responsible for the degradation of cytoplasmic proteins and organelles captured by the autophagosomes. After fusion and content exchange with the lysosomes, the autophagosome cargo is degraded [8]. Experimental models have confirmed the role of autophagy during muscle atrophy. For instance, oxidative stress induced by overexpression of a mutant superoxide dismutase protein (SOD^G93A^) causes muscle atrophy by activating autophagy [9]. Muscle-specific inactivation of genes encoding autophagy-related proteins demonstrate the essential role of autophagy in muscle homeostasis in mice [10]. In addition, myofiber degeneration is associated with complete inhibition of autophagosome formation [11, 12]. Nevertheless, an exaggerated increase in autophagy also impairs myofiber homeostasis by excessive removal of cellular components needed for normal activities, indicating that unbalanced autophagy can be a pathogenic mechanism in muscle diseases [10]. Protein synthesis and degradation in skeletal muscle are largely regulated by the mammalian target of rapamycin (mTOR) as part of the complex mTORC1 [13]. mTOR regulates many fundamental cell processes, including negative modulation of the autophagy process [14]. The positive regulation of mTOR pathway may be involved in muscle protection in SMA pathology [15, 16].

Previous findings in SMA models suggested an increase of autophagy in lower MNs [17, 18] and the regulation of SMN protein levels by autophagy modulators [19, 20]. Based on these findings it has been proposed the inhibition of autophagy as a therapeutic approach in SMA [21]. To further study the interaction between SMN deficiency and autophagy pathways, we analyzed autophagy markers and mTOR phosphorylation (Ser2448) in SMA muscle, fibroblasts and MNs. Human SMA muscle biopsies and cultured fibroblasts had reduced levels of the autophagosome indicator LC3-II. We observed decreased levels of LC3-II, Beclin 1 and p62/SQSTM1 in gastrocnemius samples from postnatal day 2 SMA mice, suggesting reduced autophagosome formation and increased autophagic flux. Nevertheless, LC3-II level was increased in human differentiated SMA MNs. On the other hand, mTOR phosphorylation at Ser2448 was reduced in SMA gastrocnemius and fibroblasts, but significantly increased in isolated MNs from mouse and human SMA models. In summary, non-neuronal and neuronal cells from SMA models displayed different molecular signs of autophagy and mTOR. Improved understanding of non-neuronal and skeletal muscle alterations in SMA will help to advance our understanding of SMA pathogenesis and the development of novel therapeutic strategies based on combinatorial treatments.

## Materials and methods

### SMA animals

Experiments involved the severe SMA mouse model FVB·Cg-Tg (SMN2)89AhmbSmn1tm1Msd/J (mutSMA). MutSMA mice (Smn−/−; SMN2+/+ were obtained by crossing heterozygous animals. Littermates mutSMA and WT (Smn+/+; SMN2+/+) were used for the experiments.

A piece of the tail from neonatal offspring was collected for genotyping. The REDExtract-N-Amp Tissue PCR Kit (Sigma) was used for genomic DNA extraction and PCR setup, with the following primers: WT forward 5′-CTCCGGGATATTGGGATTG-3′, SMA reverse 5′-GGTAACGCCAGGGTTTTCC-3′ and WT reverse 5′- TTTCTTCTGGCTGTGCCTTT-3′. Birth was defined as postnatal day 0 (P0); P2 and P5 animals were used for the experiments. All procedures were done in accordance with the Spanish Council on Animal Care guidelines and approved by the University of Lleida Advisory Committee on Animal Services (CEEA02- 01/17).

### Human samples

Subjects (or legal guardians) were given oral and written information about the experimental procedures and they provided written informed consent. All protocols were approved by Hospital Vall d’Hebron (Barcelona) and Hospital de la Santa Creu i Sant Pau (Barcelona) in agreement with their Ethics Committee guidelines.

SMA was diagnosed using the criteria outlined by the International SMA Consortium [22] and confirmed by detection of molecular alterations in the *SMN1* gene. *SMN1* genotype and *SMN2* copy number were determined as previously described [23, 24]. Human muscle samples were acquired from the Paediatric Neurology laboratory collection at Vall d´Hebron Hospital (Spanish Biobank Registry, reference C.0003146). A normal muscle sample was obtained from a 2-month-old girl undergoing surgery and a control (non-SMA) sample from a 4-month-old girl diagnosed with Pompe disease. The SMA muscle biopsies were obtained from type I and type II patients (quadriceps muscle, 4-month-old female, two copies of *SMN2*) and paraspinal muscle, 12-year-old male, two copies *SMN2*), respectively. Samples were snap-frozen and homogenized in lysis buffer. All samples were processed in parallel. In SMA type I (male, two copies of SMN2) and a healthy child (male), fibroblasts and EBV-immortalized lymphoblasts were cultured according to standard protocols [25].

### Human fibroblast cell lines culture

Cell lines were obtained from the Coriell Institute for Medical Research (Camden, NJ, USA). The Coriell Cell Repository maintains the consent and privacy of the donor samples. All cell lines and culture protocols in the present study were carried out under institutional review board guidelines at University of Lleida and the IRBLleida research center. Two human fibroblast cell lines from patients with SMA (GM03813, SMAII; and GM09677, SMAI) and one unaffected control (GM03814, Control) were purchased and cultured following manufacturer instructions. Cells were maintained in Eagle's Minimum Essential Medium (MEM) (Sigma) supplemented with non-inactivated fetal bovine serum (FBS; Gibco) (15% v/v), 0.5 M of L-Glutamine (Gibco), non-essential amino acids (Gibco) (1% v/v), and 20 μg/ml Penicillin–Streptomycin (Gibco). Cells were subcultured every 3–4 days. For western blot analysis, cells were plated at 3000–4000 cells/cm^2^ in 35 mm tissue-culture dishes and maintained in supplemented MEM. Two days later, total cell lysates were collected and submitted to western blot analysis. For immunofluorescence experiments, 5,000 cells/well were plated on 4-well dishes with collagen-coated 1 cm^2^ glass coverslips, maintained in the MEM for 24 h, then fixed in 4% paraformaldehyde in PBS.

### Differentiation of human-induced pluripotent stem cells (iPSCs) to MNs

Human iPSCs were purchased from Coriell Institute for Medical Research. The GM23411*B iPSC cell line (healthy non-fetal tissue) was the control and GM23240*B iPSC cell line (SMA) was from a patient with SMA type II (SMN2 2 copies; delta exon7-8 in SMN1). Control and SMA cells were differentiated to MNs as described [26], with minor modifications [27]. Briefly, human iPSCs were cultured on a layer of irradiated mouse embryonic fibroblasts (MEFs) (Gibco) and neuroepithelial and motoneuron progenitors (MNPs) were generated following the protocol. To induce MN differentiation, MNPs were detached with Accutase and cultured in suspension in MN induction medium (NEPIM plus 0.5 μM retinoic acid, 0.1 μM purmorphamine). Medium was changed alternate days. After six days the neurospheres were dissociated and plated on laminin-coated plates in MN maturation medium (MN induction medium supplemented with 0.1 μM Compound E [Sigma], and 20 ng/ml ciliary neurotrophic factor [CNTF], and 20 ng/ml Insulin-like growth factor 1 [IGF-1], [both from Peprotech]). Dissociated neurospheres were plated in laminin-coated four-well tissue culture dishes (Nunc, Thermo Fisher Scientific) for western blot analysis (60,000 cells/well). For immunofluorescence experiments, cells were plated on 1 cm^2^ laminin-coated glass coverslips placed into the four-well dishes (15,000 cells/well).

### Western blot analysis

Western blots were performed as previously described [28]. Spinal cord and gastrocnemius tissue samples were disaggregated using Direct Quant 100ST Buffer (DireCt Quant) and a G50 Tissue Grinder (Coyote Bioscience). Total cell lysates of cultured cells or tissue homogenates were resolved in SDS polyacrylamide gels and transferred onto polyvinylidene difluoride Immobilon-P transfer membrane filters (Millipore), using an Amersham Biosciences semidry Trans-blot (Buckinghamshire, UK). The membranes were blotted with anti-SMN (1:5000; Cat. No. 610646, BD Biosciences), anti-LC3 (1:1000; Cat. No. 2775), anti-BECLIN-1 (1:1000; Cat. No. 3738), anti-p62/SQSTM1 (1:1000; Cat. No. 5114), anti-LAMP-1 (1:1000; Cat. No. 3243), anti-p-mTOR (1:1000; Cat. No. 5536) all from Cell Signaling Technology). To control the specific protein content per lane, membranes were reprobed with anti-CypA (1:10,000; Cat. No. BML-SA296-0100, Enzolifesciences) or monoclonal anti-α-tubulin antibody (1:50,000; Cat. No. T5168, Sigma). Blots were developed using LuminataTM ForteWestern HRP Substrate (Millipore).

### Immunofluorescence

Gastrocnemius muscles from WT and mutSMA (P2 and P5) mice were dissected and fixed in 4% paraformaldehyde (Sigma) for 24 h. Tissue samples were cryoprotected with 30% sucrose in PB for an additional 48 h and finally embedded in Tissue Freezing Medium (Electron Microscopy Sciences). Sections of 16 μm-thickness were obtained in a cryostat (Leica CM3000). To break protein cross-links and unmask the antigens and epitopes, tissue sections were incubated at 450 Watts for 15 min in 10 mM Citrate Buffer solution. Cultured fibroblasts and human MNs were fixed with 4% paraformaldehyde (Sigma) for 10 min, then with cold methanol (Sigma) for 30 s.

Tissue sections and fixed cells were permeabilized with 0.2% Triton X-100 and incubated for 2 h with 5% bovine serum albumin (BSA) in PBS. Primary antibody (anti-LC3, 1:100, Cat. No. 2775; anti-beta-III-tubulin, 1:400, Cat. No. 5568, both from Cell Signaling Technology; anti-SMN 1:100; Cat. No. 610646, BD Bioscience; anti-Laminin2, 1:75, Cat. No. L0663, Sigma; anti-HB9, 1:75, Cat. No. ab92606; anti-ChAT, 1:100, Cat. No. ab18736, both from Abcam; or anti-Islet1/2, 1:50, Cat. No. 39.4D5, Developmental Studies Hybridoma Bank) was diluted in 0.2% Triton-X-100 and incubated overnight with 5% BSA in PBS. After washing, the secondary antibody was added: anti-mouse ALEXA555, 1:400, Cat. No. A21422; anti-rabbit ALEXA488, 1:400, Cat. No. A11008 (both from Invitrogen); Cy™3 AffiniPure F(ab')_2_ Fragment Donkey anti-Rat IgG (H + L), 1:400, Cat. No. 712-166-153; Cy™3 AffiniPure F(ab')_2_ Fragment Donkey anti-Sheep IgG (H + L), 1:400, Cat. No. 713-166-147 (both from Jackson ImmunoResearch). Hoechst (1:400, Sigma) staining was performed to identify nuclear localization in cell soma. Samples were mounted using Mowiol (Calbiochem) medium. Microscopy observations were performed in a FV10i Olympus confocal microscope (Tokyo, Japan). Quantification of fluorescence was performed blinded, using the NIH ImageJ software [29]. For LC3 puncta measures, the area of each cell or muscle fiber was selected and threshold level of the digital images was evenly adjusted to highlight all the spots. Quantification of the number of spots in the selected area was performed automatically using the “Find Maxima” tool.

### Statistical analysis

All experiments were performed at least three independent times. Values were expressed as mean ± estimated standard error of the mean (SEM). Statistical analysis was done with GraphPad Prism, version 8 (graphPad Software Inc). Differences between groups were assessed by two-tailed Student *t*-test or one-way ANOVA with Tuckey’s multiple comparisons or Dunnett’s multiple comparisons test for all other analysis. Values were considered significant when *p* < 0.05.

## Results

### LC3-II autophagosome marker is decreased in SMA muscle and fibroblasts, and increased in human SMA MNs

**Fig. 1:**
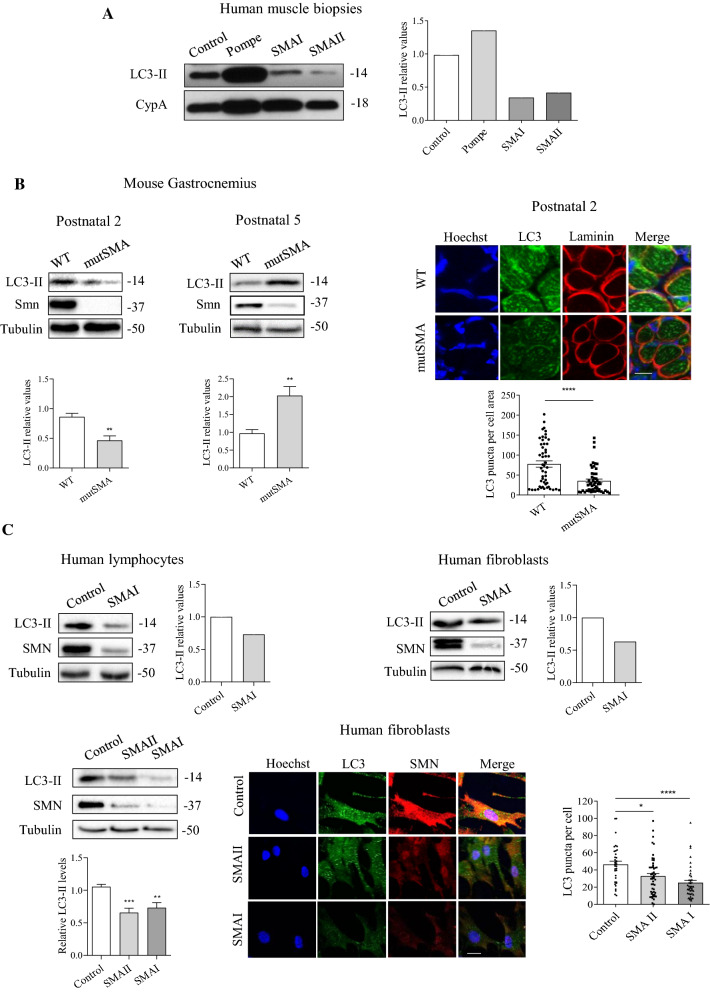
LC3-II protein level in human and murine samples from SMA muscle biopsies, and human lymphoblast and fibroblast cell lines. **A** Muscle biopsies from Control, Pompe and SMA type I and SMA type II patients were disaggregated and protein extracts were submitted to western blot analysis using anti-LC3 antibody. Membranes were reprobed with anti-CypA antibody, used as a loading control. Graph values represent the expression of LC3-II vs CypA. **B** Total cell lysates of gastrocnemius from genotyped WT and mutSMA P2 (left) and P5 (right) mice were submitted to western blot analysis using anti-LC3 and anti-SMN antibodies. Membranes were reprobed using an antibody against α-tubulin. Graph values represent the expression of LC3-II vs α-tubulin at P2 and P5, and correspond to the quantification of three independent experiments ± SEM. Asterisks indicate significant differences using Student *t* test (**p < 0.01). **B** Representative immunofluorescence images of gastrocnemius sections of P2 WT and P2 mutSMA mice using an anti-LC3 (green) and an anti-Laminin (red) antibodies. Hoechst dye (blue) was used to identify nuclei. Scale bar, 20 μm. Graphs represent the mean of LC3 positive puncta measured in WT and mutSMA myofibers, corresponding to the quantification of tree independent experiments ± SEM. Asterisks indicate significant differences using Student *t* Test (****p < 0.0001). **C** Protein extracts from fibroblast and lymphoblast cell lines were submitted to western blot analysis using anti-LC3 and anti-SMN antibodies. Membranes were reprobed with anti-α-tubulin antibody, used as a loading control. Graph values represent the expression of LC3-II versus α-tubulin. **C** Control (unaffected) and SMA II and SMA I patient fibroblast cell lines were plated and maintained in supplemented MEM. Forty-eight hours after plating, cell lysates were obtained and submitted to western blot using anti-LC3 and anti-SMN antibodies. Membranes were reprobed with an anti-α-tubulin antibody. Graph values represent the expression of LC3-II versus α-tubulin and correspond to the quantification of six independent experiments. Asterisks indicate significant differences using one-way ANOVA with Tukey’s multiple comparisons post-test (***p < 0.001, **p < 0.001). Representative immunofluorescence images of 2-day cultured Control, SMA II, and SMA I fibroblasts using anti-LC3 (green), anti-SMN (red) antibodies and Hoechst staining (blue). Hoechst was used to identify fibroblast nuclei. Scale bar, 25 µm. Graph represents the mean of LC3 positive puncta per cell and corresponds to the quantification of three independent experiments ± SEM. Asterisks indicate significant differences using one-way Anova with Tuckey’s multiple comparisons post-test (*p < 0.05; ****p < 0.0001)

LC3-II isoform is incorporated to the autophagosome membrane and the levels reflect the number of autophagosomes [30]. Western blot analysis of protein extracts obtained from SMA patients muscle biopsies (SMAI, SMA type I; and SMAII, SMA type II) showed reduced levels of LC3-II compared to non-affected control (Fig. 1A). As expected, LC3-II was increased in protein extracts obtained from muscle biopsy of a patient with Pompe disease, a well-known lysosomal storage disorder causing massive accumulation of autophagosomes [31]. To further analyze the autophagy process in SMA muscle tissue, we dissected gastrocnemius muscles [32] from the SMA mouse model FVB·Cg-Tg (SMN2) 89AhmbSmn1tm1Msd/j. Wild-type (WT) and mutant (mutSMA) genotyped mice at pre-symptomatic P2 and at disease end-point P5 [33] were used for the experiments. Protein extracts were obtained and submitted to western blot analysis using anti-LC3 antibody. LC3-II level was significantly reduced in mutSMA (0.46 ± 0.07, *p* = 0.0034) condition compared to WT control at P2 stage. In contrast, LC3-II protein was significantly increased in P5 extracts from mutSMA (2.03 ± 0.25, *p* = 0.0051) compared to the control. To validate western blot observations, the number of autophagosomes per myotube area of WT and mutSMA P2 gastrocnemius was explored by immunofluorescence using an anti-LC3 antibody and analyzed with the NIH ImageJ software as described [19]. The number of LC3 puncta per myotube area was significantly reduced in mutSMA (35.43 ± 4.63, *p* < 0.0001) compared to WT control (77.67 ± 7.84) (Fig. 1B).

To study changes in LC3-II levels in other SMA cellular models, patient SMA lymphoblast and SMA fibroblast cell lines were analyzed. Protein extracts were obtained from 2-day cultured SMA lymphoblast or fibroblast samples (kindly provided by Dr. Eduardo Tizzano, Hospital Santa Creu i Sant Pau, Barcelona) and submitted to western blot analysis using anti-LC3 antibody. LC3-II protein level was clearly reduced in both cellular types, compared to controls (Fig. 1C). Additionally, primary myoblasts obtained from the paraspinal muscle biopsy in SMA type II (3 copies of *SMN2*) showed reduced LC3-II levels (data not shown). Likewise, total protein cell lysates of 2-day cultured SMAII and SMAI fibroblasts (from Coriell Institute; see Materials and Methods) were analyzed. Results showed that LC3-II protein level was significantly decreased in SMA fibroblasts (SMAII 0.65 ± 0.06, *p* = 0.0006; and SMAI 0.73 ± 0.08, *p* = 0.004) compared to the clinically unaffected control (Fig. 1C). To further analyze changes in the number of autophagosome compartments in SMA fibroblasts, 1-day cultures were processed for immunofluorescence analysis of LC3 protein. Cells were fixed and LC3 immunostaining was performed with anti-LC3 antibody. The number of fluorescent LC3 puncta was quantified using NIH ImageJ software. The area of each fibroblast was selected and LC3 puncta were counted per cell. Fluorescent puncta in SMA fibroblasts (SMAII 32.76 ± 3.01, *p* = 0.0104; and SMAI 24.97 ± 2.96, *p* < 0.0001) were significantly reduced compared to the control (46.37 ± 3.76) (Fig. 1C).

**Fig. 2:**
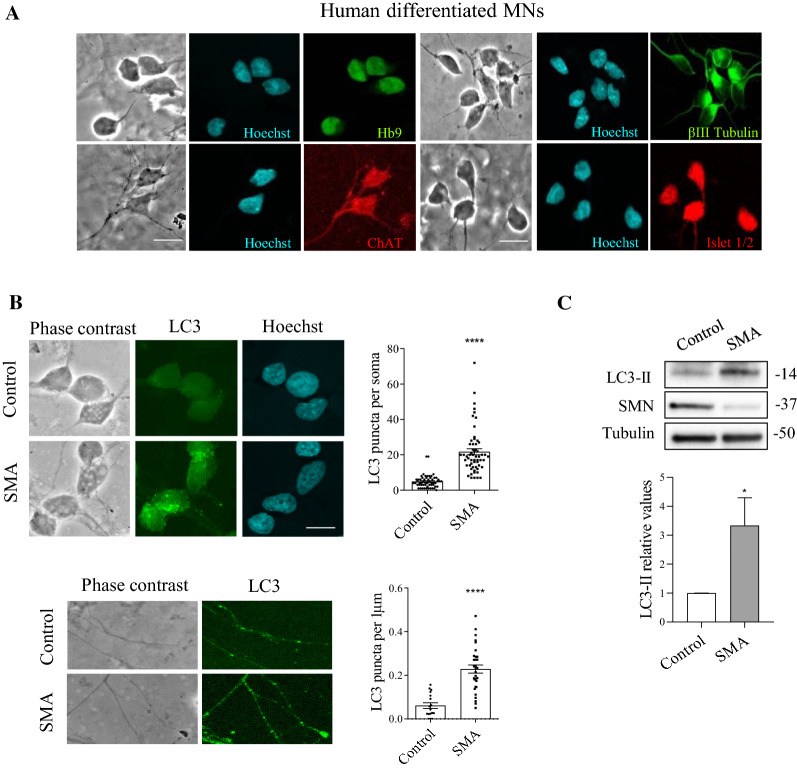
Increased levels of LC3-II in human SMA differentiated MNs. **A** Representative phase contrast and immunofluorescence images of 7-day differentiated Control and SMA human MNs, showing the MN markers HB9 (green left section), ChAT (red left section), Islet 1/2 (red right section) and beta-III-tubulin (green right section). Hoechst (blue) staining was used to identify MN nuclei. Scale bar 15 μm. **B** Representative immunofluorescence confocal images of 7-day differentiated human MNs using an anti-LC3 (green) antibody. Hoechst (blue) dye was used to identify nuclei. Graphs represent the mean of LC3 positive puncta per soma (above) or per neurite (1 μm) (below) and correspond to the quantification of three independent experiments ± SEM. Asterisks indicate significant differences using Student *t* test (****p < 0.0001). Scale bar, 15 µm. **C** Protein extracts of 7-day differentiated control and SMA human MNs were submitted to western blot using an anti-LC3 antibody. Membranes were reprobed with anti-α-tubulin antibody. Graph values represent the expression of LC3-II versus α-tubulin and correspond to the quantification of six independent experiments ± SEM. Asterisks indicate significant differences using Student *t* test (*p < 0.05)

SMA and non-affected control human iPSC cells (from Coriell Institute, see Materials and Methods) were differentiated to MNs following the protocol described [26, 27] (Fig. 2A). We examined LC3 level in six days differentiated SMA and control MNs. Since MNs are highly polarized cells, we chose immunofluorescence to observe LC3 protein in cell soma and neurites. After differentiation, cultures were fixed and processed using anti-LC3 antibody. The number of LC3 puncta was quantified using NIH ImageJ software. Results showed a significant increase of LC3 in soma and neurites of human SMA differentiated MNs (soma, 21.72 ± 1.67 puncta per soma, *p* < 0.0001; neurites, 0.22 ± 0.018 puncta per µm, *p* < 0.0001) compared to the unaffected control (soma, 4.87 ± 0.47 puncta per soma; neurites, 0.06 ± 0.012 puncta per µm) (Fig. 2B). Protein extracts of 7-day differentiated human SMA and control MNs were submitted to western blot analysis using an anti-LC3 antibody. LC3-II level was increased in human SMA (3.33 ± 0.96, *p* = 0.035) MNs compared to control condition (Fig. 2C). Together these results indicated an increase of LC3-II autophagosome marker in mouse isolated and human differentiated SMA MNs.

### Beclin 1, p62/SQTM1 and LAMP-1 protein level are altered in SMA gastrocnemius and human SMA fibroblasts

**Fig. 3:**
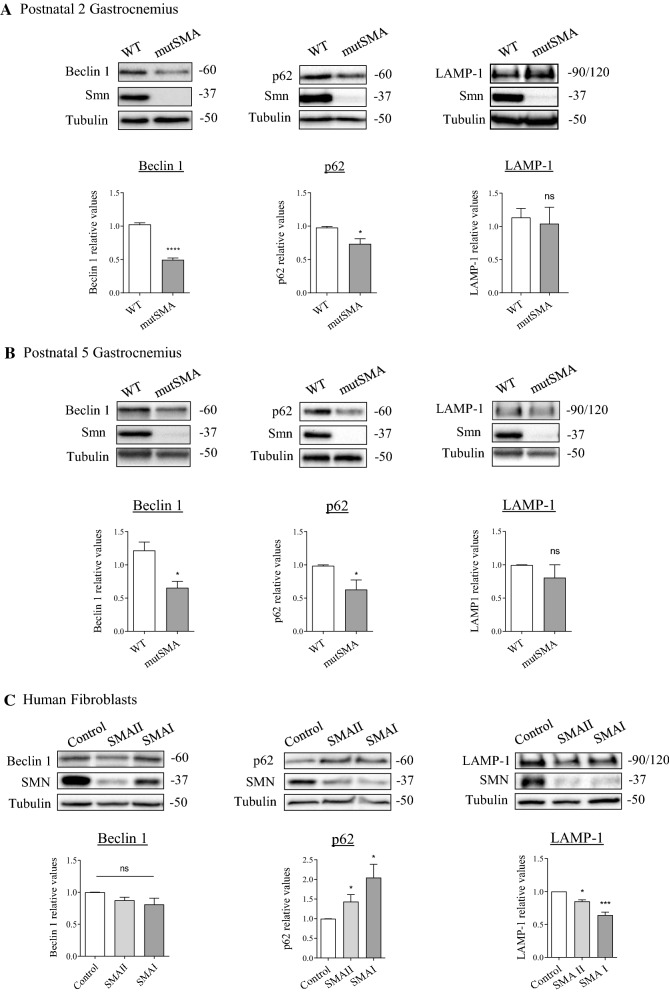
Beclin 1, p62/SQTM1 and LAMP-1 protein levels in SMA gastrocnemius and human SMA fibroblast cell lines. Total cell lysates of gastrocnemius from P2 (**A**) and P5 (**B**) genotyped WT and mutSMA mice were submitted to western blot analysis using anti-Beclin 1, anti-p62/SQSTM1 or anti-LAMP-1, and anti-SMN antibodies. Membranes were reprobed using an antibody against α-tubulin. Graph values represent the expression of the targeted proteins vs α-tubulin at P2 and P5, and correspond to the quantification of four and three independent experiments ± SEM, respectively. Asterisks indicate significant differences using Student *t* test (*p < 0.05, ****p < 0.0001, no significant differences (ns) *p* > 0.05). **C** Control (unaffected) and SMA II and SMA I patient fibroblast cell lines were maintained in the presence of supplemented MEM. Forty-eight hours after plating, cell lysates were obtained and submitted to western blot analysis using anti-Beclin 1, anti-p62/SQSTM1 or anti-LAMP-1, and anti-SMN antibodies. Membranes were reprobed with an anti-α-tubulin antibody. Graph values represent the expression of the targeted proteins versus α-tubulin and correspond to the quantification of three independents experiments ± SEM. Asterisks indicate significant differences using one-way Anova with Tuckey’s multiple comparisons post-test (*p < 0.05, ***p < 0.0005, no significant differences (ns) *p* > 0.05)

To further explore changes in SMA muscle and fibroblasts autophagy pathway, we next analyzed the levels of Beclin 1, p62/SQTM1 (p62) and LAMP-1 proteins. Beclin 1 activity controls the assembly of the double-membrane autophagosome and regulates the number of these structures and LC3-II level [34]. Protein extracts of WT and mutSMA P2 and P5 genotyped mice were submitted to western blot analysis using an anti-Beclin1 antibody. Beclin 1 protein level was significantly reduced in both SMA P2 (0.49 ± 0.028, *p* < 0.0001) and SMA P5 (0.65 ± 0.098, *p* = 0.022) conditions compared to the P2 and P5 WT controls (Fig. 3A, B). To evaluate whether Beclin 1 was also reduced in human SMA fibroblast cell lines, protein extracts from 2-day cultured control, SMAII, and SMAI fibroblasts (Coriell Institute) were submitted to western blot analysis using an anti-Beclin 1 antibody. No significant differences between control and SMA Beclin 1 protein levels were observed, although the level was slightly reduced in SMA conditions (SMAII 0.87 ± 0.047 and SMAI 0.81 ± 0.097) (Fig. 3C).

p62/SQTM1 protein (p62) is degraded by autophagy and links ubiquitinated proteins to the autophagic machinery to enable their degradation in the lysosome [35, 36]. Accumulation of p62 indicates reduced autophagic flux. p62 protein level was significantly reduced in both P2 mutSMA (0.73 ± 0.078, *p* = 0.022) and P5 mutSMA 0.62 ± 0.14, *p* = 0.049) compared to the respective WT controls (Fig. 3A, B), suggesting increased autophagic flux in SMA gastrocnemius. In contrast, western blot analysis of protein extracts from 2-day cultured human control and SMA fibroblasts revealed significantly increased p62 in SMA (SMAII 1.43 ± 0.17, *p* = 0.014; and SMAI 2.04 ± 0.33, *p* = 0.01) compared to control, suggesting reduced autophagic flux in these cells (Fig. 3C).

To evaluate whether alteration of the autophagic flux in muscle cells and fibroblasts is associated to changes in the lysosomal compartment, we analyzed the level of lysosome associated membrane protein-1 (LAMP-1), one of the major protein components of the lysosomal membrane [37]. LAMP-1 protein level was not statistically different in SMA P2 (1.04 ± 0.24, *p* = 0.75) and P5 (0.805 ± 0.19, *p* = 0.43) mouse gastrocnemius compared to the respective WT control conditions (Fig. 3A, B). Conversely, LAMP-1 was significantly reduced in human SMA fibroblasts (SMAII 0.85 ± 0.02, *p* = 0.03; SMAI 0.64 ± 0.04, *p* = 0.0004) compared with non-affected control (Fig. 3C), indicating that lysosomal compartment may be altered in these cells.

### mTOR phosphorylation at Ser2448 is reduced in SMA muscle and increased in cultured SMA MNs

**Fig. 4:**
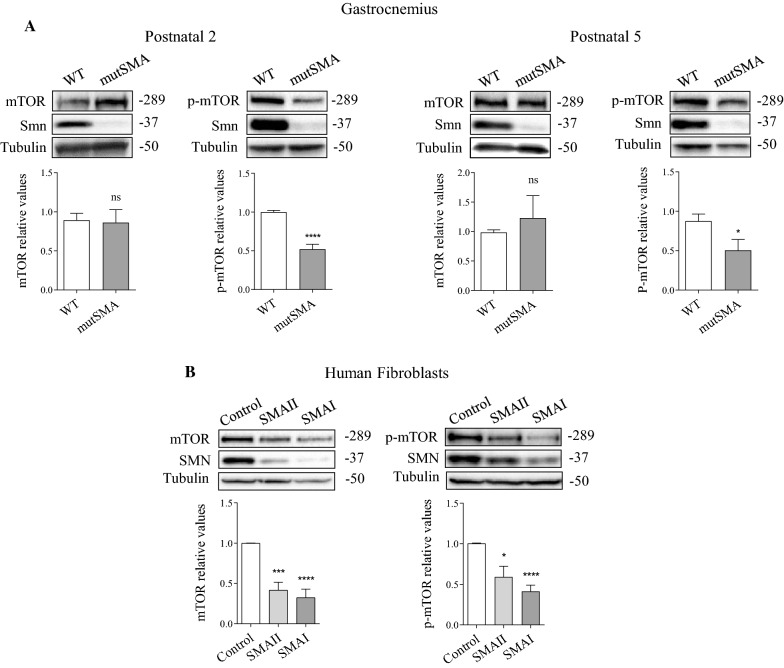
Changes of mTOR protein level and phosphorylation at Ser2448 in protein extracts of SMA tissues and human SMA fibroblast cell lines. **A** Total cell lysates of P2 and P5 gastrocnemius from WT and mutSMA genotyped mice were submitted to western blot analysis using anti-mTOR, anti-phospho-mTOR(Ser2448) and anti-SMN antibodies. Membranes were reprobed using an anti-α-tubulin antibody. Graph values represent the expression of mTOR or phospho-mTOR(Ser2448) (p-mTOR) versus α-tubulin and correspond to the quantification of at least five independent experiments ± SEM. Asterisks indicate significant differences using Student *t* test (*p < 0.05; ****p < 0.0001; non statistic (ns) *p* > 0.05). **B** Protein extracts from 48 h cultured control (unaffected) and SMA II and SMA I patient fibroblast cell lines were submitted to western blot using anti-mTOR or anti-phospho-mTOR(Ser2448) and anti-SMN antibodies. Membranes were reprobed with an anti-α-tubulin antibody. Graph values represent the expression of mTOR or p-mTOR versus α-tubulin and correspond to the quantification of five independent experiments ± SEM. Asterisks indicate significant differences using one-way Anova with Dunnett’s multiple comparisons post-test (***p < 0.001; ****p < 0.0001)

mTORC1 signaling is a well-known negative regulator of the autophagy pathway and muscle atrophy has been related to the reduction of mTOR phosphorylation at Ser2448 [38]. In this context, we examined mTOR protein level and Ser2448 phosphorylation in SMA models. Protein extracts from genotyped WT and mutSMA P2 and P5 mouse gastrocnemius were submitted to western blot analysis using anti-mTOR antibody or anti-mTOR (phospho Ser2448) antibody. No differences of mTOR protein level were observed in WT and mutSMA samples at P2 and P5. Nevertheless, mTOR Ser2448 phosphorylation level was significantly reduced in P2 and P5 mutSMA (P2, 0.52 ± 0.06, *p* < 0.0001; P5, 0.504 ± 0.13, *p* = 0.037), compared to WT controls (Fig. 4A). Total protein cell lysates of control, SMAII and SMAI 2-day cultured fibroblasts were submitted to western blot analysis. mTOR protein level was significantly reduced in cell lysates from SMA fibroblasts (SMAII 0.41 ± 0.096, *p* = 0.0002; SMAI 0.32 ± 0.10, *p* < 0.0001) (Fig. 4B). As expected, the level of mTOR Ser2448 phosphorylation was reduced in SMA fibroblasts (SMAII 0.59 ± 0.13, *p* = 0.0114; SMAI 0.41 ± 0.077, *p* = 0.0009) compared to the clinically unaffected control (Fig. 4B).

**Fig. 5:**
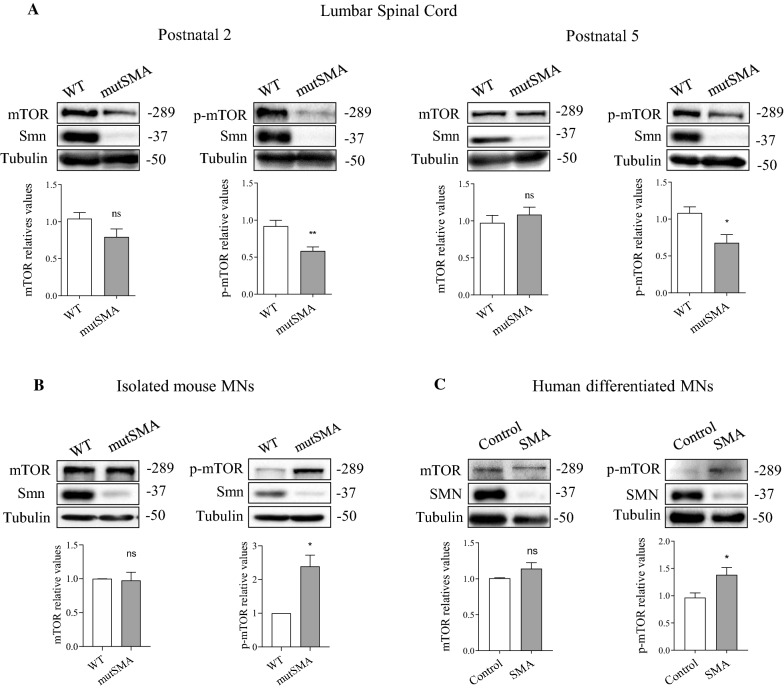
Changes of mTOR protein level and phosphorylation at Ser2448 in SMA spinal cord and isolated MNs. (A) Spinal Cords from P2 and P5 genotyped WT and mutSMA mice were dissected and protein extracts were submitted to western blot analysis using anti-mTOR or anti-phospho-mTOR(Ser2448) antibodies. Membranes were reprobed with anti-α-tubulin antibody. Graph values represent the expression of mTOR or p-mTOR versus α-tubulin and correspond to the quantification of at least five independents experiments ± SEM. Asterisks indicate significant differences using Student *t* test (*p < 0.05; **p < 0.01; no significant differences (ns) *p* > 0.05). Protein extracts of 6-day cultured WT and mutSMA mice MNs (B) and 7-day differentiated Control and SMA human MNs (C) were submitted to western blot analysis using anti anti-mTOR or anti-phospho-mTOR(Ser2448) antibodies. Membranes were reprobed with anti-α-tubulin antibody. Graph values represent the expression of mTOR or phospho-mTOR(Ser2448) (p-mTOR) versus α-tubulin and correspond to the quantification of at least four independent experiments ± SEM. Asterisks indicate significant differences using Student *t* test (*p < 0.05; no significant differences (ns) *p* > 0.05)

On the other hand, total protein extracts from P2 and P5 spinal cord lumbar fragments of genotyped WT and mutSMA mouse were submitted to western blot to analyze mTOR and phospho-mTOR. The mTOR protein level was not significantly modified in WT and mutSMA conditions. Nevertheless, phospho-mTOR at Ser2448 was significantly reduced in P2 and P5 mutSMA (P2, 0.58 ± 0.054, *p* = 0.006; P5, 0.67 ± 0.11, *p* = 0.026) conditions compared to P2 and P5 WT controls, respectively (Fig. 5A). To further explore whether mTOR phosphorylation at Ser2448 was altered in isolated MNs, we analyzed mTOR protein and phospho-Ser2448-mTOR in cell lysates from mouse and human cultured MNs. MNs were obtained from spinal cords of genotyped WT and mutSMA mouse embryos (E13.5) [28]. After 6 days in vitro, total cell lysates were collected and submitted to western blot. Protein level of mTOR was not significantly modified in mutSMA MNs compared to the WT condition. However, phospho-mTOR analysis revealed a significant increase of Ser2448 phosphorylation in mutSMA (2.39 ± 0.33, *p* = 0.0125) cultures compared to WT controls (Fig. 5B). Protein extracts of seven days differentiated human SMA and control MNs were obtained and submitted to western blot analysis. The mTOR protein level did not show significant differences between SMA and control MNs. However, mTOR phosphorylation at Ser2448 was increased in SMA (1.38 ± 0.13, *p* = 0.0198) condition compared to the control (Fig. 5C).

## Discussion

In the present work, we analyzed proteins related to the autophagy process in several SMA models, including muscle tissue. Degeneration and loss of function of spinal cord MNs and muscle denervation are two of the pathological hallmarks of SMA. How SMN depletion leads to MN degeneration is not fully understood and remains the focus of intense research. Recent advances have highlighted the involvement of other tissues in the pathophysiology of SMA and skeletal muscle appears to be an important candidate [7, 39] Therefore, the analysis of muscle contribution to neurodegeneration has acquired particular importance and further studies in muscle collapse might lead to new strategies in SMA therapy. Several pathways trigger muscular atrophy, amongst which autophagy deregulation has a primary role [40].

Two main findings emerged from our study: the autophagy process was significantly altered in SMA muscle cells; and mTOR and autophagy pathways alterations had different profiles in SMA muscle, fibroblasts, and MNs. The reduced levels of the autophagosome marker LC3-II in muscle, lymphocytes, and fibroblasts indicate either decreased autophagosomes formation or increased autophagy flux. In contrast, at the disease end-point (P5 gastrocnemius) LC3-II was clearly increased in SMA condition, indicating augmented autophagosomes in muscle cells at the final stage of the disease when cells are likely collapsed. Previous results suggested that skeletal muscle atrophy in severe SMA mouse is marked by increased proteasomal degradation but not by autophagosomal protein breakdown [33]. However, our results indicated that autophagy markers, including LC3, Beclin 1 and p62/SQSTM1, are deregulated in SMA muscle. In this context, some evidence indicates that proteasome and autophagy activity are compromised in Smn-reduced MNs [17, 19, 41, 42]. Treatment with the autophagy inhibitor Baphilomycin A1 reduces Smn protein level in MNs and the inhibition of the proteasome activity reverts this effect [19]. These results indicate that both autophagy and proteasome regulate Smn protein level in SMA neurons. In terms of muscle tissue, the atrophy process may be exacerbated when both pathways are altered [43] and muscle-specific regulatory mechanisms could make the scenario more complex [7, 44].

Beclin 1 is a BH3-only domain autophagy protein that regulates autophagy and membrane trafficking involved in several physiological and pathological processes. It can mediate at every major step in autophagic pathways, from autophagosome formation to autophagosome/endosome maturation [34]. In the present work, we described a significant reduction in Beclin 1 protein level in mouse SMA gastrocnemius. Beclin 1 reduction may contribute to the slowdown/decrease of autophagosome formation and lowering LC3-II levels [34]. Beclin 1 is the substrate of several proteases including caspases and calpain [45, 46]. It is known that caspase and calpain pathways are deregulated in Smn-reduced cells in SMA pathology [27, 47, 48]; therefore, the activation of these pathways in muscle cells could be the basis for a decrease in Beclin 1. Studies of muscle-specific SMN reduction may help to elucidate the contribution of autophagy, apoptosis, and calpain pathways on SMA muscle atrophy.

**Fig. 6:**
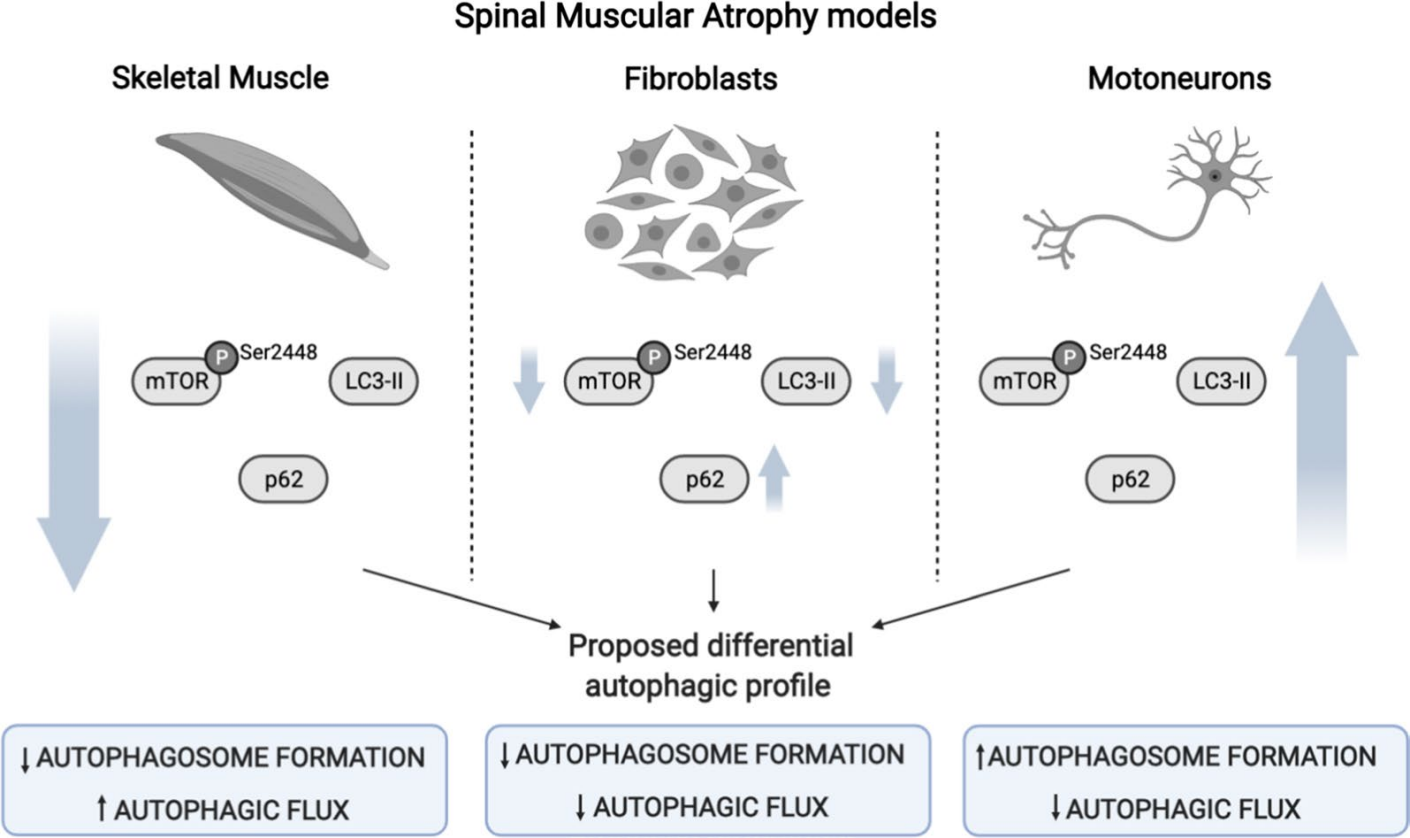
Schematic representation of the differences in autophagy-related proteins expression and mTOR phosphorylation occurring in Spinal Muscular Atrophy disease models. SMA skeletal muscle reveals an autophagy profile compatible with reduced autophagosome formation and increased autophagic flux. Nevertheless, human SMA fibroblast cell lines evidence reduced autophagosome formation and reduced autophagic flux. Finally, the results in SMA MNs suggest an increase of autophagosome formation and reduced autophagic flux (figure created with www.BioRender.com)

Based on our analysis, we developed a hypothesis that autophagy deregulation in SMA cells could be subject to the cell type. For instance, p62/SQSTM1 protein which is a well-known indicator of autophagic flux modifications [35, 36], is significantly reduced in SMA gastrocnemius suggesting an increase in the autophagic flux. Interestingly, in SMA fibroblasts western blot analysis revealed an increase of p62/SQSTM1, indicating a reduced autophagic flux in these cells. These observations, together with our results showing no differences in Beclin 1 level in SMA fibroblasts (Fig. 3B) and autophagy analysis in SMA MNs and spinal cord, supported this hypothesis. LC3-II level (Fig. 6), Beclin 1, and p62/SQSTM1 are increased in SMA MNs [17, 19, 21] further reinforcing the hypothesis suggesting differences in the autophagy process between SMA muscle cells and SMA MNs. Therefore, it would be worthwhile to include the regulation of the autophagy pathway as a complementary treatment strategy for SMA disease; however, tissue type autophagy changes should be considered in developing this therapeutic approach.

mTOR is mostly known for its role in cell proliferation and growth, but is also involved in additional cellular functions such as autophagy [49]. mTOR research has revealed the enormous complexity of its signaling network in mammalian cells. Early studies of mTOR signaling revealed that mTORC1 activation is associated with muscle hypertrophy [50]. However, chronic mTORC1 activation in the muscle also results in severe muscle atrophy, primarily due to an inability to induce autophagy in this tissue [13, 40]. Considering that turnover of old or damaged tissue plays a critical role in muscle growth, these outcomes suggest that alternating periods of high and low mTORC1 activity are essential for maintaining optimal muscle health and function [13]. Atrophy and hypertrophy of skeletal muscle are associated with decreased and increased in Ser2448 phosphorylation, suggesting that modulation of this site may have an important role in the control of protein synthesis [38]. Our results indicate reduced Ser2448 phosphorylation in SMA mouse gastrocnemius with no modifications of mTOR protein level. The activation of mTOR is also essential for neuromuscular junction (NMJ) maintenance and its inhibition causes NMJ loss and triggers a dying-back process producing MN injury [51]. For instance, it has been recently published that SMA muscle regulates mTOR dependent axonal local translation via the secreted molecule CTRP3, compromising axonal outgrowth and protein synthesis in SMA neurons [52]. Our results showed that mTOR phosphorylation in isolated SMA mouse and human MNs was significantly increased in these cells, but was reduced in protein extracts from spinal cords. Spinal cord lysates include MNs and their surrounding cells, therefore, reduced mTOR phosphorylation indicates a generalized decrease in the spinal cord. These observations may suggest a differential regulation of mTOR phosphorylation in MNs and non-neuronal cells in the context of SMA. In addition, mTOR inhibition by rapamycin is deleterious for SMA mice [21] and for SOD1 (G93A) ALS mice [53], but some autophagy inductors have a beneficial effect on ALS [54–56]. However, it should be noted that mTOR inhibition in SMA and ALS models may have a differential effect on autophagy and mTOR functions in muscle and MNs. For instance, different responses to autophagy induction have been described in muscle and nervous system in a Huntington disease mouse model treated with the rapamycin homolog everolimus [57].

## Conclusions

The present study describes modified autophagy-related proteins in SMA muscle tissue. The analysis of mouse SMA gastrocnemius revealed an autophagy profile compatible with reduced autophagosome formation and increased autophagic flux. However, our results and previous SMA MNs studies [17, 19, 21] suggest an increase of autophagosome formation and reduced autophagic flux (Table 1 and Fig. 6). The third type of SMA cells analyzed, human fibroblasts cell lines, evidenced a different autophagy pattern with reduced autophagosome marker and increased p62/SQSTM1. Additionally, mTOR protein level and Ser2448 phosphorylation were dissimilar in the SMA cells analyzed, mTOR level was not modified in gastrocnemius and MNs but was reduced in fibroblast, and Ser2448 phosphorylation was reduced in muscle cells and increased in MNs. Our observations indicate that autophagy and mTOR deregulation differ between SMA cell types (Fig. 6), suggesting a need to consider such differences before using autophagy modulators as combinatorial therapies for SMA treatment. Additionally, current SMA treatments might modify the tissue specific autophagy response. Hence, to further explore these effects could provide new insights into the SMA therapy field.

**Table 1:** Representation of autophagic markers protein level amongst different human and mouse Spinal Muscular Atrophy models

Autophagy marker	Protein level	SMA cell type and tissue	References
LC3-II	Decreased	Human: muscle, lymphocytes, fibroblasts Mouse: muscle	Present work
	Increased	Human: differentiated MNs Mouse: spinal cord, isolated MNs	Present work [17, 21, 27]
Beclin 1	Decreased	Mouse: muscle	Present work
	Increased	Mouse: spinal cord, isolated MNs	[17, 21]
p62/SQSTM1	No change	Mouse: spinal cord	[21]
	Increased	Human: differentiated MNs, fibroblasts Mouse: isolated MNs	[18–20]
	Decreased	Mouse: muscle	Present work
LAMP-1	No change	Mouse: muscle	Present work
	Increased	Human: fibroblasts	Present work
mTOR	No change	Human: differentiated MNs Mouse: muscle, spinal cord, isolated MNs	Present work
	Decreased	Human: fibroblasts	Present work
p-mTOR (Ser2448)	Decreased	Human: fibroblasts Mouse: muscle, spinal cord	Present work [16]
	Increased	Human: differentiated MNs Mouse: isolated MNs	Present work

## Data Availability

All data generated or analyzed during this study are included in this published article.

## Abbreviations

SMN: Human Survival Motor Neuron protein
*SMN1* and * SMN2*: Human Survival Motor Neuron 1 and Survival Motor Neuron 2 genes, respectively
Smn: Mouse Survival Motor Neuron protein
*Smn*: Mouse Survival Motor Neuron gene
SMA: Spinal Muscular Atrophy
MN: Motoneurons
mTOR: Mammalian target of rapamycin
iPSCs: Induced pluripotent stem cells
NBMc: Neurobasal medium complete
NEP: Neuroepithelial cells
NEPIM: Neuroepithelial induction medium
MNP: Motoneuron progenitors
